# From cane to nano: advanced nanomaterials derived from sugarcane products with insights into their synthesis and applications

**DOI:** 10.1080/14686996.2024.2393568

**Published:** 2024-08-19

**Authors:** Bhavya Mod, Arun V. Baskar, Rohan Bahadur, Ehsan Tavakkoli, Lukas Van Zwieten, Gurwinder Singh, Ajayan Vinu

**Affiliations:** a Global Innovative Centre for Advanced Nanomaterials, College of Engineering, Science and Environment (CESE), School of Engineering, The University of Newcastle, Callaghan, NSW, Australia; b School of Agriculture, Food and Wine, The University of Adelaide, Glen Osmond, SA, Australia; c NSW Department of Primary Industries, Wollongbar Primary Industries Institute, Wollongbar, NSW, Australia

**Keywords:** Sugarcane, silica nanoparticles, nanocomposite, capping agent, nanocellulose, nano biochar, 102 porous, nanoporous, nanostructured materials, 103 composites, 104 carbon and related materials 308 materials resources, recycling

## Abstract

Sugarcane-based products are inherently rich in elements such as silicon, carbon and nitrogen. As such, these become ideal precursors for utilization in a wide array of application fields. One of the appealing areas is to transform them into nanomaterials of high interest that can be employed in several prominent applications. Among nanomaterials, sugarcane products based on silica nanoparticles (SNPs), carbon dots (CDs), metal/metal oxide-based NPs, nanocellulose, cellulose nanofibers (CNFs), and nano biochar are becoming increasingly reported. Through manipulation of the experimental conditions and choosing suitable starting precursors and elements, it is possible to devise these nanomaterials with highly desired properties suited for specific applications. The current review presents the findings from the recent literature wherein an effort has been made to convey new development in the field of sugarcane-based products for the synthesis of the above-mentioned nanomaterials. Various nanomaterials were systematically discussed in terms of their synthesis and application perspectives. Wherever possible, a comparative analysis was carried out to highlight the potential of sugarcane products for the intended purpose as compared to other biomass-based materials. This review is expected to stand out in delivering an up-to-date survey of the literature and provide readers with necessary directions for future research.

## IMPACT STATEMENT

This review focuses on sugarcane-derived nanomaterials such as silica, nano cellulose, nanofibers, nanocrystals and metal/nonmetal nanoparticles and their application in various energy and environmental fields.

## GRAPHICAL ABSTRACT

**Figure:**
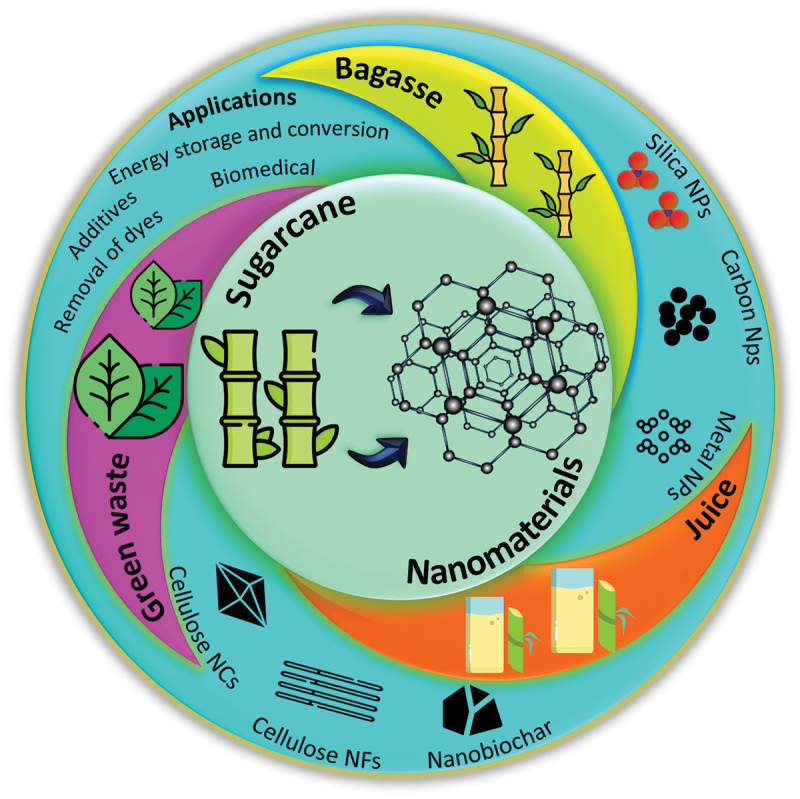

## 1. Introduction

Sugarcane, a member of the *Poaceae* family, commonly referred to as grass, is a highly versatile crop utilized for the production of various commodities including sugar, ethanol, molasses, biofuel, and livestock fodder. Cultivating sugarcane requires a sub-tropical climate and ample water availability, leading to its predominant cultivation in equatorial regions [1]. By 2030, Brazil and India are projected to contribute 38% and 27%, respectively, to the global production share, highlighting their significant roles in the sugarcane industry [2]. The extraction of sugarcane juice, a vital step in processing, yields numerous by-products, with bagasse being the most prominent. Bagasse, a dry, fibrous residue remaining after juice extraction, finds versatile applications in energy production, paper manufacturing, and packaging materials [3]. Additionally, other by-products such as molasses, filter muds, mill ash, and green trash are utilized in various sectors, including fertilizer production [4]. Ethanol is another useful product that is derived from sugarcane as it consists of sugar in the form of disaccharides. Following the invention of ethanol combustion engines, there has been an exponential increase in sugarcane cultivation for producing ethanol. Biomass-derived ethanol is increasingly viewed as a viable option to reduce our dependencies on fossil fuels [5]. This has, however, led to the generation of large volumes of sugarcane solid and liquid waste estimated at over 279 million tons annually across the world [6]. The sheer scale of these agro-industrial wastes has become a huge waste management challenge and hence requires sustainable pathways to address this issue. One of the promising approaches to tackle this problem is to utilize these agro wastes for the development of nanomaterials that find use in a variety of applications [7,8]. Therefore, such a strategy is useful in the conversion of waste products for the sugarcane industry into highly useful products.

Nanotechnology has been a defining scientific trend in recent times. Nanomaterials are materials where the constituent units have dimensions at the nanoscale, i.e. <100 nm [9]. Their small size and high surface area equip them with unique physico-chemical properties, different from their bulk forms [10]. Today, nanomaterials find applications in almost every technological field, most notably being used in adsorption and separation [11–17], electronics [18], energy storage and conversion [19], catalysis [20–28], sensing [29–32], drug delivery [33–36], and agriculture [37]. While much of the research is focussed on exploring new applications, there is an equal need to devise innovative and sustainable methods for nanomaterial synthesis and functionalisation [38]. Most conventional methods for the synthesis of nanomaterials such as electrospinning, templating, and sputtering employ expensive, toxic chemicals, even involving heavy metals, which are harmful and unsustainable, both environmentally and economically [39]. As an alternative to this, the term ‘Green synthesis’ is gaining ground as a sustainable means of nanomaterial production [40]. The colloquial term refers to an environmentally benign process of synthesizing materials using natural materials as starting precursors and/or process agents. Such techniques have also emerged as important tools for waste management in various industries [41].

Despite their wide presence, the categorisation of nanomaterials remains fluidic with arbitrary classifications based on their physical and chemical nature. In this review, we make use of the most common nanomaterial categorisation to better understand the different types of nanomaterials that can be derived from biomass sources [17] which include nanoparticles (NPs), nanofibers, nanocrystals, nanocomposites and nanochars. The large volume and varieties of sugarcane-based waste materials make them ideal candidates for their utilization in various green synthesis techniques to produce nanomaterials. Recently, several works have employed sugarcane waste products to produce different nanomaterials [42]. For example, bagasse-derived silica nanoparticles (SNPs) [43], carbon black nanoparticles [44], metal oxide carbon nanocomposites [45], metal oxide supported on cellulose nanocrystals [46], and sugarcane juice derived spinel ferrite [47], and TiO_2_ [48] NPs are reported. There is also considerable research being undertaken in applying sugarcane green waste-derived nanocarbons for material and agricultural applications [49]. Several other potential applications of sugarcane products are also being actively researched with great promise.

**Figure 1.**
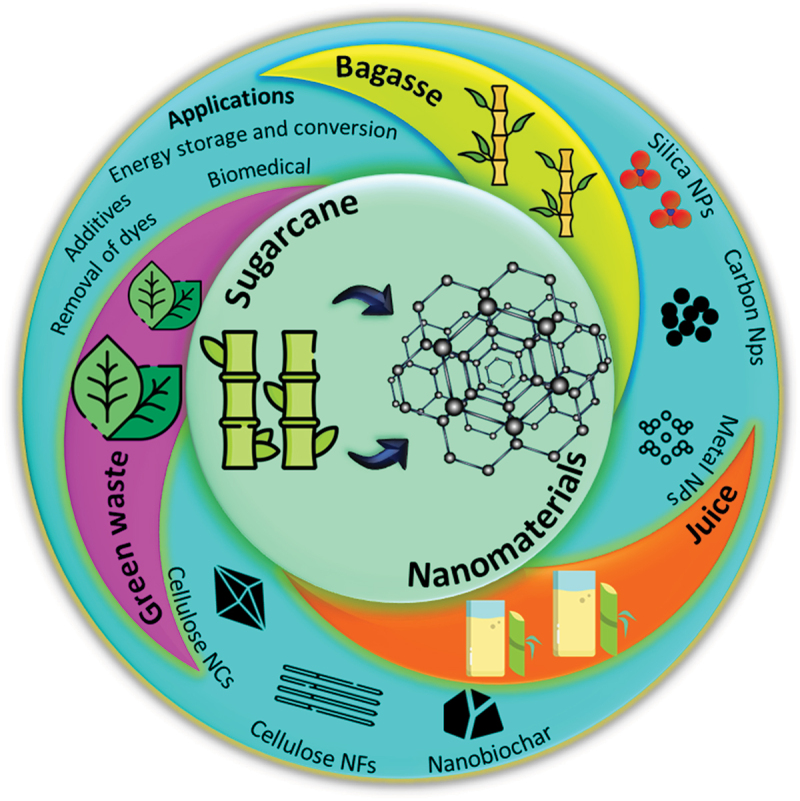
Schematic representation of application of sugarcane products for the synthesis of nanomaterials.

In line with the growing research interest, various review articles have tried to group different potential aspects of sugarcane products. For instance, however, to the best of our knowledge, no published literature has aimed to synergise the various synthesis and applications of sugarcane products vis-a-vis nanotechnology. For instance, Seroka et al. [50], September et al. [51], and Prabha et al. [52] reviewed SNPs from sugarcane products, whereas Torgbo et al. [53] went on to illustrate the cellulosic-based materials from sugarcane bagasse. However, our review is different to these previously existing reviews, as we have attempted to comprehensively review the green synthesis of a thorough range of nanoparticles including SNPs, metal species, carbon-based NPS nanocellulose, nanofibers, and nanobiochar-based nanoparticles from sugarcane products and their functionalisation, which makes it unique and attractive for the aspiring researchers working in this field. Along with the updated survey of the literature, a comparative analysis, wherever possible, of sugarcane-based derived nanomaterials with other biomass materials was also illustrated. The discussions in each section and sub-section were thoroughly analysed and concluding remarks were added. The recent advancements and the key challenges identified through the comprehensive survey of literature in this review open up new avenues for the utilization of sugarcane-derived nanomaterials in various application fields. It will serve as a useful piece of information for researchers to explore innovative concepts and develop sustainable and cost-effective nanomaterials. Figure 1 presents an overview of the production of different kinds of nanomaterials from various agro products obtained from the sugarcane plant.

## 2. Nanoparticles (NPs) derived from sugarcane-based products

Sugarcane products have been used in many studies, either by themselves or in addition to other agro materials to produce different NPs. In the majority of the studies referred, bagasse is used as a precursor, while sugarcane juice is used as a chemical catalyst or a controlling agent. The use of sugarcane leaves for nanomaterial synthesis has also been successfully demonstrated. A variety of NPs including silica, carbon, and metal species could be synthesized using sugarcane products, and these are discussed in detail in the forthcoming sections. A summary of different nanomaterials derived from bagasse and sugarcane juice is presented in Table 1.

**Table 1.** Sugarcane product-derived NPs, their properties, and application perspectives.

Sugarcane material	Nanoparticle	Synthesis method	Notable properties of NPs	Application/performance	Ref.
Bagasse	Silica	Sol-gel method Thermal method	High purity, amorphous, micro-structured SNPs (95%)	Ceramics, glasses, and other construction fields	[54]
Leaf	Silica	Sol-gel method Thermal method	High purity, amorphous, micro-structured SNPs (95%)	Ceramics, glasses, and other construction fields	[54]
Bagasse	Silica	Magnesio-thermic method	Amorphous SiO_2_ with 97% purity	Optical and electronics industry	[55]
Bagasse	Silica	Thermal method	SNPs with biogenic properties	Biomedical field	[55]
Bagasse	Silica	Sol-gel method	High purity SNPs (98%)	Sustainable source for industrial production	[56]
Bagasse	Silica	Metallothermic reaction	3D nanoporous SNPs	Clean energy and energy storage	[57]
Bagasse	Silica	Thermal method	Biocompatible SNPs	Biomedical industry	[43]
Bagasse	Silica	Thermal method	Biocompatible mesoporous and core-shell SNPs	Supercapacitor, bioimaging, drug delivery, biosensors	[52]
Bagasse	Silica	Sol-gel	Amorphous, high-adsorption SNPs	Dye adsorption and environmental remediation	[58]
Bagasse	Silica	Freeze-dried	SNPs with improved surface area and porosity	Rubber industry	[59]
Bagasse	Carbon dot	Chemical oxidation	Fluorescent, biocompatible and high quantum yield	Biosensor, bioimaging, and drug delivery	[60]
Bagasse	Carbon dot	Freeze drying	Monodispersed, enhanced photoluminescence, high photostability and biocompatibility	Biomedical and photoelectronic	[61]
Bagasse	Graphene oxide	Calcination	Cost-effective, sustainable method to produce graphene oxide from bagasse	Dye adsorption and treatment of wastewater	[62]
Bagasse	Graphene oxide	Calcination	Cost-effective, sustainable method to produce GO from bagasse	Gas sensors, energy storage, and other functional devices	[63]
Sugarcane juice	Cupric oxide	Juice-mediated stabilisation	Small-sized nanoparticles using sugarcane juice as a capping and complexing agent.	Anti-microbial applications	[64]
Sugarcane juice	Cupric oxide	Juice-mediated stabilisation	Urchin-like nanoparticles using sugarcane juice as a capping and complexing agent	Catalyst, peroxidase, and enzyme mimetic activity	[65]
Sugarcane juice	Zinc oxide	Combustion route	Green, surfactant-free and sustainable approach towards the genesis of cuprous oxide (Cu_2_O) nanoparticles	Dye degradation, environmental remediation, and photocatalysis	[66]
Sugarcane juice	Cadmium ferrite	Combustion route	Synthesized CdFe NPs exhibited excellent photocatalytic activity	Dye degradation and textile waste remediation	[67]
Sugarcane juice	Zinc ferrite	Combustion route	Synthesis of narrow band gap ZF nanoparticles with photocatalytic potential to degrade organic and mixed dyes	Applications in anti-microbial and photocatalytic functions	[68]
Sugarcane juice	Silver doped silver chloride	Capping agent, halide supplying agent	Synthesis of hybrid Ag@AgCl plasmonic nanoparticles (NPs)	Photocatalytic applications	[69]
Sugarcane juice	Silver	Capping agent	Synthesis of bio-composite between spherical silver nanoparticles and chitosan	Antifungal properties	[70]
Sugarcane juice	Silver	Capping agent	Sustainable synthesis. Anti-fungal properties	Anti-microbial applications	[71]
Sugarcane juice	Carbon dot	Capping agent	Synthesis of graphitic carbon nitride (g-C_3_N_4_) composites (CD/g-C_3_N_4_) for dye degradation	Photocatalyst, environment remediation	[72]

### 2.1. Silica nanoparticles (SNPs)

The use of sugarcane-derived products, especially bagasse, is being explored for synthesizing several non-metallic NPs with the most prominent being SNPs [43]. Sugarcane has microscopic phytolith structures that are essentially silica particles that provide structural strength to the tissues [59]. These phytoliths can be rich sources for silica extraction from sugarcane plants. SNPs possess exceptional physical, chemical, electronic, and optical properties, with a high surface area and small particle size [73].

**Figure 2.**
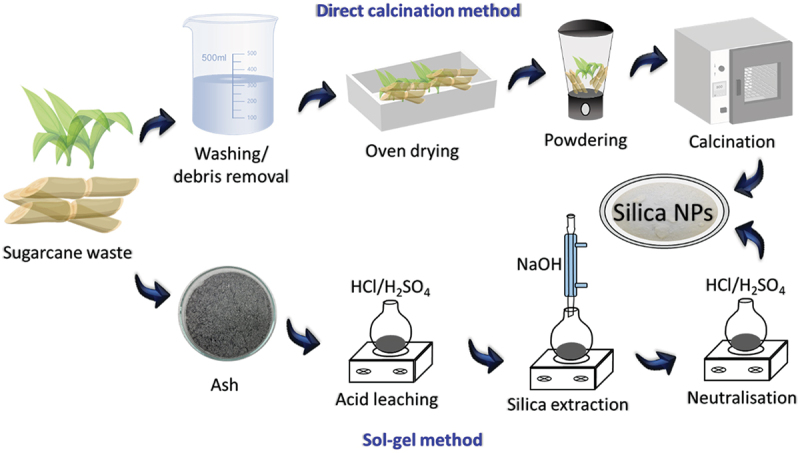
Direct calcination and sol-gel methods for the synthesis of SNPs from sugarcane waste products.

Several works have utilized sugarcane bagasse for the synthesis of SNPs. There are two main methods found in the majority of literature. The first is the ‘direct calcination’ method, where, as the name suggests, bagasse/ash powder is directly calcinated to form SNPs [74]. The second technique is the ‘sol-gel method’. Here, a strong base such as sodium silicate is used to extract silica and the removal of silicon occurs in the form of silicates, which leads to the formation of gel under controlled pH conditions. An important application-based distinction between the two is that while calcination produces biocompatible, biogenic SNPs, the NPs synthesized by the sol–gel method lose their biogenic properties and therefore have limited use in life science fields. Figure 2 shows the details of these two prominent methods used for the synthesis of SNPs from sugarcane-based products. For the synthesis of SNPs, the first step, common across all works, is the initial washing and milling of bagasse which is required to remove basic physical impurities as well as to create a more homogenous texture for the subsequent reactions. Acid-leaching is another purification step seen in a majority of referred publications, wherein, the bagasse/bagasse ash powder is leached using a strong acid, such as HCl or H_2_SO_4_. Pressurised thermal conditions (using an autoclave) have also been used to increase the efficacy of this reaction. The purpose of this step is to remove all metal ion impurities (Na, Ca, Mg, Fe, K, Mn, and Al) and also to initiate hydrolysis of the structural organic components including lignin, cellulose, and hemicellulose [59]. Following this, the material is thoroughly cleansed with distilled water and dried at 80 °C. The step is also important in determining the crystallinity and surface area of the synthesized SNPs [57].

**Figure 3.**
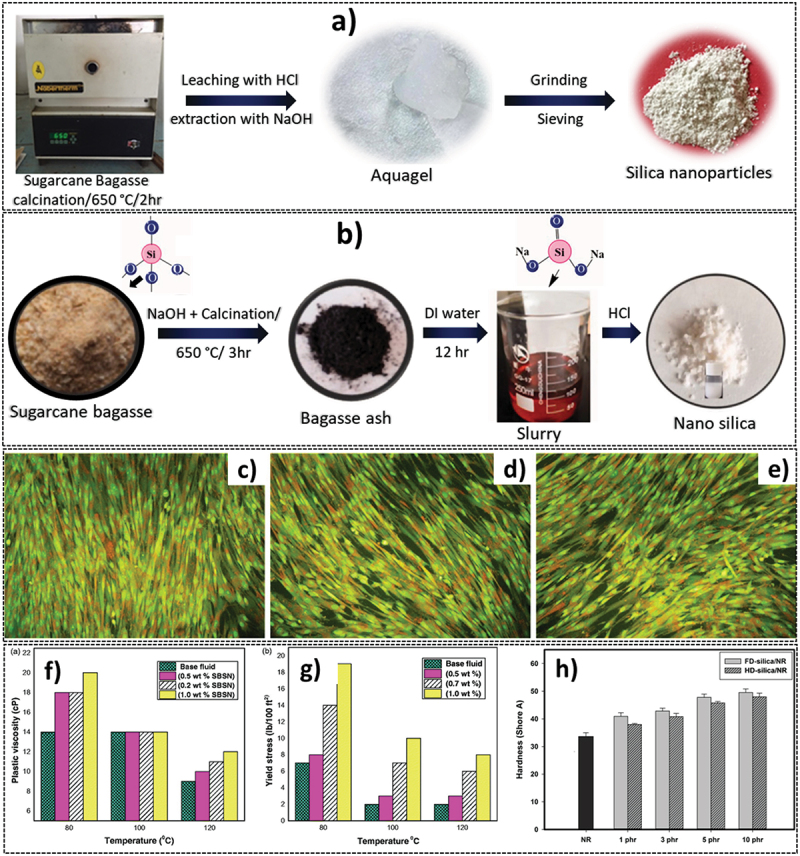
a) synthesis of SNPs from sugarcane bagasse using the sol-gel method, reproduced with permission [55]. Copyright 2019, Elsevier, and b) synthesis of mesoporous nano silica from co-calcination of a mixture of sugarcane bagasse and NaOH and the sol-gel operation, reproduced with permission [75] copyright 2021, Taylor & Francis, c-e) effect of biogenic silica nanoparticles (BSNPs) on the morphology of WI-38 cells in control, and 50 μg/mL and 200 μg/mL dosing of BSNPs, indicating that morphology does not undergo any significant changes, reproduced with permission [43] copyright 2017, Wiley, f & g) changes in plastic viscosity and yield stress of drilling fluid incorporated with sugarcane bagasse silica nanoparticles (SBSNs) as a result of varied content of SBSNs and the temperature reproduced with permission [75] copyright 2021, Taylor & Francis, h) increasing trend in the hardness of natural rubber (NR) and its composites in the freeze-dried (FD) and heat dried (HD) forms with SNPs reproduced with permission [59]. Copyright 2020, Springer.

Teixeria et al. made a comparative study among three different calcination-based routes to synthesize SNPs using both sugarcane bagasse and sugarcane leaf as silica precursors. Following the initial washing, the raw material in the first route was further pressed into a finer powder before undergoing calcination. In the second route, after milling, the powder was subjected to an acid-leaching step before calcination. Finally, in the third route, as a slight change from the second, the acid-leaching step was positioned to instead succeed the calcination step. The study concludes that the leaching step in either succession improves silica content in both bagasse (95%) and leaf (88%) ashes while also enhancing the total surface area of SNPs compared to a direct calcination synthesis [54]. Falk et al. carried out a similar study comparing two methods to synthesise SNPs, i.e. sol-gel method vs direct calcination of bagasse ash followed by leaching [55]. In the sol-gel method, the bagasse ash was first leached with HCl and then it was added to an NaOH solution to form sodium silicate. The formed solution was then subjected to a ‘gelling stage’ by titration through HCl (Figure 3a). The obtained xerogels were then filtered, dried, and characterised. The SNPs obtained via the sol–gel process exhibited a higher purity, uniformity (size averaging around 10 nm) and amorphous character in comparison to the conventional calcination method, while the latter was more useful in preserving the ‘biogenic’ nature of the synthesized SNPs with less uniformity in size, shape, and distribution. The sol–gel method has also been used in several other published literature owing to the ease of production and homogenous nature of the synthesized SNPs [55].

Hamad et al. in their study synthesized SNPs from sugarcane bagasse for use as an additive to industrial drilling fluids for enhancing their properties. This was achieved through the thermal treatment method using a muffle furnace followed by the sol–gel method for the synthesis of nano-silica as shown in Figure 3b. They also confirmed the biodegradable nature of the synthesized particles [75]. The properties of the obtained SNPs were similar to those reported by Rovani et al. where the authors had managed to synthesise high-purity SNPs with high surface area and a particle size of >20 nm [76].

Vaibhav et al. made a comparative study to determine the potential of various agricultural wastes in producing SNPs [56]. Extraction of silica was done from four sources, namely, rice husk, bamboo leaves, sugarcane bagasse, and groundnut shell. After removing the moisture, each of the samples was incinerated at 900 °C, followed by the dissolution of silica through alkali leaching using 1 M NaOH. Then, the solution was filtered and precipitated using H_2_SO_4_ with a subsequent drying process. The resultant silica was highly pure in all samples except for groundnut. With a relatively high yield of 71%, sugarcane bagasse was found to be one of the best agricultural sources for the production of high-grade SNPs [56]. In another report, Praneetha et al. demonstrated a hybrid technique for the extraction of SNPs with an extra added step involving microwave-assisted metallothermic reaction to enhance their porosity and crystallinity [57]. In their study, after the initial acid-leaching treatment, the raw material was heated in a microwave muffle furnace at 650 °C for 20 minutes to obtain amorphous SNPs. After annealing, this powder was mixed with Mg powder and microwaved at 650 °C for another 20 minutes for reducing SNPs into crystalline silicon with the interconnected three-dimensional porous network. Finally, the annealed samples were dissolved in a solution (HCl, H_2_O, and ethanol) to remove impurities and nanoporous silica was extracted. The authors also demonstrated that the derived nanoporous silica is an excellent platform for the synthesis of various hybrid materials with carbon, multi-walled carbon nanotubes (MWCNTs), and graphene for enhancing the conductivity and delithiation capacities [57]. The study by Khotseng et al. showed the synthesis of crystalline SNPs of sizes under 30 nm from bagasse ash using l-cysteine hydrochloride monohydrate acid and tetrapropylammonium hydroxide for acid treatment and extraction, respectively [77].

In terms of application, SNPs (biogenic) are most widely used in the biomedical sector for various processes including tissue engineering, regenerative medicine, drug delivery, protein adsorption, scaffolds, molecular imaging, and gene therapy. This has generated much interest, with a growing number of literatures dedicated towards their synthesis and potential uses. For example, Athinarayanan et al. synthesized biogenic SNPs through a thermal method using bagasse as a source material [43]. The ‘biocompatibility’ of these SNPs was tested on human lung fibroblast cells (hLFCs) WI-38 through MTT assay along with testing for cellular morphology, ROS levels, cell cycle progression, and gene expression. They also reported the effect of calcination temperature on the crystallinity of the SNPs wherein it was observed that the SNPs prepared at lower calcination temperatures showed broader peaks indicative of the amorphous nature of the compound, while a more crystalline structure was observed when it was prepared at higher calcination temperature [43]. Jambhrunkar et al., in their studies on different cell lines including HeLa and HCT-116, reported that SNPs at a lower concentration (200 μg/ml) did not alter cellular morphology or affect cell viability [78]. Athinarayanan et al., on the other hand, proposed an even higher upper limit of 400 μg/mL, which can be safely used for biomedical applications if the exposure time is increased, and that exposure of 200 μg/mL SNPs did not affect the morphology of the hLFC cells (Figures 3d–e). A longer exposure time to SNPs also contributed to a slight reduction of ROS levels and an up-regulation of CYP1A gene [43].

Apart from their conventional applications in the above-mentioned fields, researchers have also been discovering the use of SNPs in improving the efficacy of other processes as possible additives. The addition of SNPs even in small proportions can substantially improve the properties like viscosity, yield stress and filter cake thickness of drilling fluids (DF) highlighting their potential as a cheap, sustainable additive as suggested by Hamad et al. [75]. Water-based mud is the most commonly used DF owing to its cheap cost and environmentally benign nature. These, however, face a few problems with respect to density, drag, stability, and torque increase. To mitigate this, SNPs as additives have been proposed. The interactions between SNPs and the DF matrix were shown to significantly enhance the viscosity (SNPs added in three different concentrations of 0.5, 0.2, and 0.1 wt %) and yield stress (SNPs added in three different concentrations of 0.5, 0.7 and 1.0 wt %) (Figures 3f,g) while simultaneously reducing filtration loss, filter cake permeability and filter cake thickness. This was largely due to the small size of the SNPs that provided a higher surface area for chemical interaction and a more uniform distribution of particles [75].

Similarly, Boonmee et al. explored SNPs as an additive to natural rubber (NR) while simultaneously comparing the efficiency of heat-dried (HD) SNPs and freeze-dried (FD) SNPs for the same purpose [59]. Four different filler concentrations of 1, 3, 5 and 10 phr (parts per hundred rubber) were designed for the addition of NPs. While noting that the FD SNPs were superior in various inherent properties including surface area and porosity, the authors also observed that the incorporation of these SNPs with natural rubber strongly improved the scorch time, torque, cure rate index, elongation, hardness, tear strength and tensile strength with a better performance compared to HD SNPs. For example, the increase in hardness with the incorporation of FD or HD SNPs into NR is shown in Figure 3h. It was theorized that a higher surface area and porosity of SNPs provided stronger chemical interactions, especially during the vulcanisation process, which led to the formation of chemical and physical cross-links with the rubber matrix, strongly fortified the resultant rubber composites [59]. Similar to Hamad et al., the authors in this study also noted the proportionate enhancement in most of the composite’s qualities with a higher additional concentration of SNPs. In another report, Sholeh et al. demonstrated the efficiency of the composite of rubber and various bagasse-derived SNPs with different textural parameters and morphologies, which were prepared at different temperatures and pHs as reinforcing filler materials, and found the performance to be better when higher concentration SNPs were added [79]. Sugarcane bagasse has also been deployed as a coating material for NPs to enhance their properties. For instance, Razali et al. coated magnetic Fe_3_O_4_ NPs with bagasse through co-precipitation. The use of these coated NPs was made as a low-cost adsorbent for the removal of methylene dye from wastewater samples [80]. Overall, sugarcane-based products are ideal starting precursors for the synthesis of SNPs, the properties of which can be suitably tailored by employing variation in the synthesis process.

### 2.2. Carbon-based NPs

**Figure 4.**
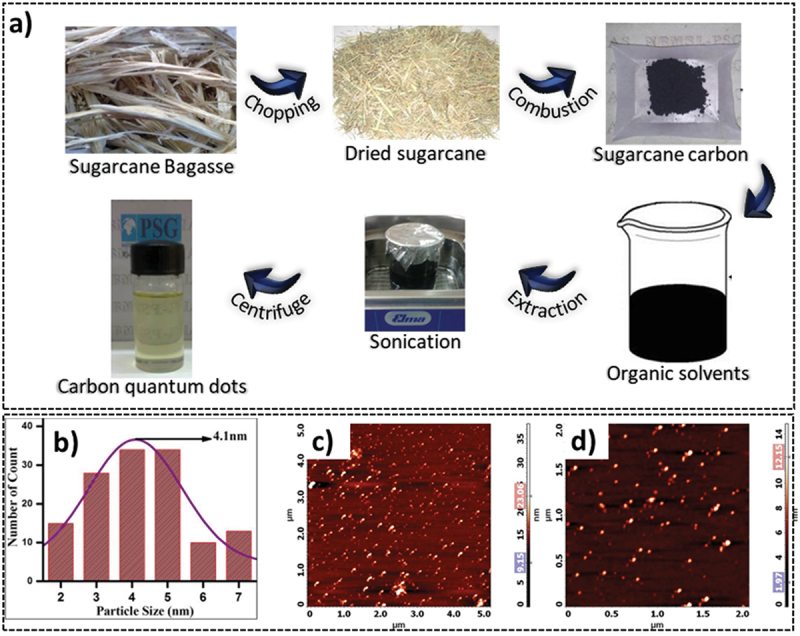
a) schematic for the synthesis of sugarcane pulp-derived CQDs, b) the particle size distribution of CQDs with a maximum particle size of 4.1 nm, and c & d) atomic force microscopic imaging confirming the 3-5 nm range for the size of CQDs reproduced with permission [60]. Copyright 2016, Elsevier.

Agro-industrial residues and biomass wastes, including sugarcane bagasse, are ideally suited for the fabrication of carbon-based nanomaterials such as carbon dots. Carbon dots (CDs) are one of the most versatile NPs which have nanocrystalline regions of sp^2^ hybridised graphitic carbon [81]. Their small size and large surface area coupled with improved photostability, high quantum yield, biocompatibility and biodegradability make them well-suited for several different applications. There are two main approaches to synthesizing CDs, namely, bottom-up and top-down. The bottom-up approach involves the use of a passivation agent to agglomerate smaller molecules into larger CDs [72]. Whilst the technique offers an advantage in terms of size and shape control, the high cost, low yield and toxicity limit its use in biomedical applications [82]. The top-down approach, on the other hand, can make use of biodegradable precursors to synthesize low-cost, biocompatible CDs. Keeping true to these two approaches, various methods have been used for the synthesis of CDs, including wet chemical, hydrothermal, electrochemical, and microwave-mediated synthesis. Thambiraj et al. made use of the chemical oxidation method to synthesize carbon quantum dots (CQDs) from sugarcane bagasse pulp through an extraction procedure developed by Zhang et al. [60,83]. The bagasse pulp was air dried and combusted at 60 °C following which the CQDs were extracted with toluene as a solvent (Figure 4a). The authors reported the resultant CQDs to have high fluorescence, stability, surface area with an average particle size of 4.1 ± 0.17 nm (Figure 4b), and a high carbon purity of 87%. The atomic force microscopy (AFM) images revealed a size between 3 and 5 nm for the CQDs (Figure 4c,d). These CQDs also contained carbonyl and hydroxyl functional groups on their surface aiding in their application as sensory and drug delivery devices [60].

Du et al. made modifications to the top-down approach to synthesizing CDs. Using bagasse as a precursor, the authors dialysed the mixture of supernatant against ultra-pure water to obtain the CD residues which were then freeze-dried. The CDs obtained were monodispersed and spherical in shape. With regard to the mechanism, the authors theorise that the bagasse particles undergo an initial hydrothermal breakage (under high temperature, high-pressure conditions) where oxidation of water leads to their breakdown into smaller products and loss of other impurities. These thenfollow a ‘bottom-up’ (again, under hydrothermal conditions) approach to re-polymerise into CDs [61]. There are other instances that reported the preparation of the CDs from sugarcane products. For example, Sim et al. made use of sugarcane juice as a precursor using the hydrothermal route to produce CDs. In the first step, the mixture of sugarcane juice and ethanol was autoclaved following which it was subjected to multiple washes (using dichloromethane and acetone) and centrifugation steps to produce CDs. These were then conjugated with graphitic carbon nitride (g-C_3_N_4_) to form a novel nanocomposite for bisphenol A (BPA) dye degradation under natural sunlight [72]. The performance of the nanocomposites was 3.5-fold higher than that of the pristine g-C_3_N_4_, confirming the beneficial role of CDs for enhancing the performance in BPA degradation.

Other than CDs, graphene oxide (GO) could also be derived from sugarcane products. For instance, Hashmi et al. devised a simple, single-step synthesis for GO from three agro-waste products including sugarcane bagasse, orange peels, and rice bran and compared their properties. The carbonaceous precursors were mixed with ferrocene and then heated in a muffle furnace at 300 °C to obtain GO NPs from the respective source materials. The authors reported an average particle size of 4 nm with bagasse and around 2.5 nm when used in a composite with two other agro residues, making it a cheap, environmentally benign alternative for GO nanomaterial synthesis [84]. A similar synthesis of GO from bagasse was also demonstrated by Somnathan et al. who used simple oxidation in a muffle furnace with ferrocene catalyst [63].

### 2.3. Metal and metal oxide NPs

While sugarcane bagasse is an excellent source of silica, sugarcane juice makes for an ideal catalyst for several synthesis reactions. The composition of sugarcane juice includes sucrose, a non-reducing sugar present in high proportions (between 81% and 87%), a few reducing sugars (including glucose and fructose), and other biomolecules including amino acids, organic acids, starch, and dextran. Sugarcane juice also contains trace amounts of inorganic ions (e.g. chloride, sodium and calcium) [85]. Glucose, fructose, and other reducing sugars present in sugarcane could act as capping agents, influencing the shape and size of the NPs while preventing their agglomeration, whereas sucrose could prevent premature reduction of nucleated ions, acting as a stabilising agent. Other biomolecules present in the juice provide a medium for the formation of intermediate complexes in the synthesis process. Numerous different metal/metal oxide NPs have been derived from sugarcane juice/other sugarcane productmediated [86,87] synthesis processes, which will be elaborated in the forthcoming discussion.

#### 2.3.1. Copper (Cu) based NPs

**Figure 5.**
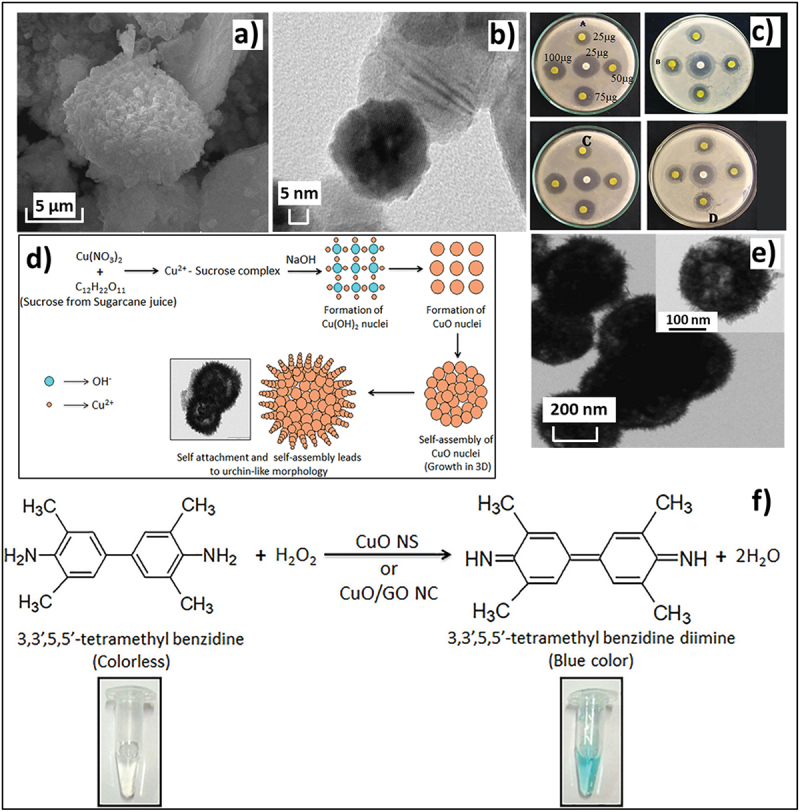
a and b) SEM and TEM images of the spherical size CuO NPs synthesized from copper nitrate by using 10 ml of sugarcane juice as a stabilising agent, c) antimicrobial activity of these CuO NPs in four doses for A) *E. coli*, B) *P. aeruginosa*, C) *S. aureus*, and D) *b. subtilis* wherein the results are comparable to those with the standard ciprofloxacin (shown in the middle) reproduced with permission [64] copyright 2019, Elsevier, d) synthesis of CuO nanospheres using sugarcane juice derived sucrose and copper nitrate, e) TEM image confirming the ~400 nm size of the CuO nanospheres, and f) color based colourimetric tmb-based H_2_O_2_ sensing using the CuO nanospheres or CUO and graphene oxide composite reproduced with permission [65] copyright 2020, Elsevier.

Copper-based NPS from sugarcane products has also been an actively explored field of research [88]. As demonstrated by Mary et al., sugarcane juice can be effectively used as a stabilising agent with the concentration tweaked to obtain CuO NPs of different sizes and shapes [64]. In their work, three different volumes of sugarcane juice (2, 5 and 10 mL) were mixed with the copper nitrate solution prior to the commencement of NP synthesis. While at lower concentrations, NPs exhibited a mixture of different shapes, a greater amount of juice addition capped the NPs into a predominantly spherical shape with an average size of 30 nm as confirmed by SEM and TEM studies (Figure 5a,b).

The synthesized NPs also exhibited good anti-bactericidal activity against *E. coli, P. aeruginosa, S. aureus*, and *b. subtilis* (Figure 5c). The zone of inhibition of these NPs was comparable to that of the reference drug Ciprofloxacin. This anti-bacterial efficacy was attributed to the capping properties of glucose and fructose present in sugarcane juice that prevented agglomeration and restricted the NPs to a size small enough to penetrate the bacterial cell membrane and induce toxicity [64].

In another report, Bhattacharjee et al. made use of different components of sugarcane juice to functionalize and provide unique properties to CuO nanospheres by using microwave-assisted synthesis [65]. The synthesis of the CuO nanospheres was achieved through crystal growth occurring via Ostwald ripening with a particle size of ~400 nm (Figure 5d,e). Various biomolecules present in the juice acted as a labile binding agent for Cu^2+^ ions prior to the formation of CuO NPs. These also got readily adsorbed on the surface of the NPs to serve as capping/stabilizing agents. The non-reducing nature of sucrose made it an ideal medium for the reaction, preventing the reduction of Cu(II), and thereby avoiding the formation of Cu_2_O. Sucrose and other cane biomolecules also influenced the crystal structure of the CuO nanospheres. A uniform crystallographic orientation was achieved owing to surficial H-bonding and π-π interactions of the coated biomolecules. The authors further investigated the enzymatic potential of the CuO/GO (graphene oxide) complex with regard to enzyme-mimetic (H_2_O_2_ sensing) and antioxidant properties (Figure 5f). Here again, the biomolecules adsorbed on CuO nanospheres play a vital linking role in forming H and π bonding with graphene atoms, enhancing the stability of the composite material. The study is an excellent example of an exploratory investigation into the many roles sugarcane juice plays in aiding the synthesis of NPs.

In a different work, the authors demonstrated the same principle to achieve a successful biosynthesis of tin oxide NPs (SnO_2_) [65]. Assisted by microwave irradiation, biomolecules from sugarcane juice formed a weak complex with the free Sn^2+^ ion derived from the SnCl_2_ precursor. Carbohydrates, amino acids, and other biomolecules were shown to prevent agglomeration of nucleated particles, capping them to form spherical SnO_2_ QDs having an average particle size of 4 nm with a tetragonal crystal structure.

#### 2.3.2. Zinc (zn) based NPs

**Figure 6.**
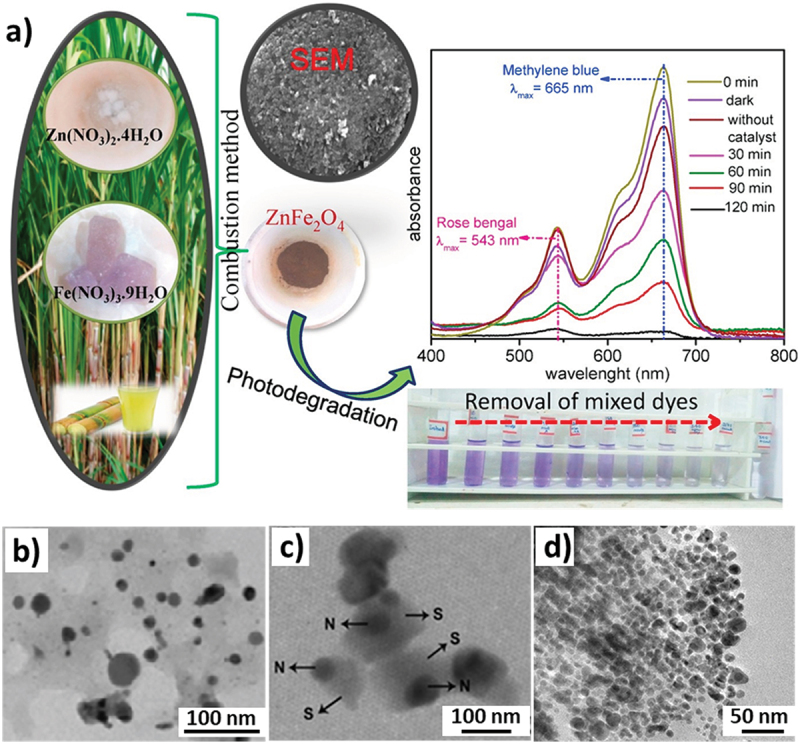
a) The synthesis of ZnFe_2_O_4_ NPs using sugarcane juice as a mediator and its application for the degradation of methylene blue and Rose Bengal, reproduced with permission [68] Copyright 2018, Elsevier, b) TEM images of Ag@AgCl nps synthesized using 30 ml of sugarcane juice demonstrating the uniform size and well dispersed, reproduced with permission [69] copyright 2014, American Chemical Society, c) sugarcane juice acting as a stabilising agent (S) for Ag NPs (N), reproduced with permission [70] copyright 2017, IOP, and d) TEM images of Ag NPs synthesized using the extract of sugarcane leaves, reproduced with permission [71], copyright 2017, Springer.

Yadav et al. synthesized ZnO NPs, demonstrating versatile applications including photocatalysis, antimicrobial, antioxidant, and enzyme-mimetic [89]. Similar studies exploring the photocatalytic properties of sugarcane-stabilized NPs were also done by Patil et al. [67,68] wherein they synthesized solar-activated cadmium ferrite (CdFe_2_O_4_) and zinc ferrite (ZnFe_2_O_4_) NPs (Figure 6a). These materials displayed a successful photocatalytic degradation of methylene blue (MB) and azo and mixed dyes (MB and Rose Bengal). The same research group also experimentally varied the juice concentration being added to the synthesis mixture to conclude that an increasing juice content led to a slight decrease in the surface area of the NPs while significantly altering the pore diameters. A similar use of sugarcane juice was also investigated by Joshi et al. to produce TiO_2_ NPs which were then doped with different metallic ions including Al^3+^, Cu^2+^, and Zn^2+^ to demonstrate their potential for magneto-optical applications [90].

#### 2.3.3. Silver (ag) based NPs

Sugarcane liquid/juice has also been employed for the synthesis of Ag-related NPs [91]. Kulkarni et al. in their work reported a novel technique for synthesizing Ag/AgCl NPs for their application as a photocatalyst. Sugarcane juice was mixed with AgNO_3_ to form AgCl NPs with sugarcane juice supplying chloride ions in the reaction. Glucose in the juice aided in the reduction of silver ions leading to the formation of Ag@AgCl NP based composite and also as a capping agent restricting the composite size to under 50 nm (Figure 6b), providing a larger surface area and thereby enhancing their property for photocatalytic degradation of MO and MB dyes [69]. Paulkumar et al. used a similar protocol to synthesize spherical Ag NPs in which sugarcane juice acted as a stabilising agent (Figure 6c) and the material was then used in a chitosan biocomposite displaying antibacterial properties against *B. subtilis* (MTCC 3053), *K. planticola*, *S. faecalis*, *P. aeruginosa*, and *E. coli* [70]. On the other hand, the moderate antifungal nature of sugarcane leaf derived Ag NPs (Figure 6d) was demonstrated by Velu et al. against pathogenic fungi including *P. capsici, C. Acutatum*, and *C. fulvum* [71]. Elemike et al. applied the same procedure to derive bare Ag NPs by mixing cane juice with the precursor. While glucose causes the reduction of Ag^+^ to Ag^0^, the OH groups in sucrose molecules are hypothesized to aid in hydrogen abstraction, stabilizing the synthesized Ag NPs [90]. Panwar et al. also made use of sugarcane juice as a reducing agent to convert AgNO_3_ to Ag_2_O NPs in an ionic polymer nanocomposite for possible applications in sensors and other electronics [91].

The above studies firmly establish a successful application of sugarcane products for the synthesis of different types of NPs. However, despite the extensive research, there yet remains a lot to be explored with respect to research on replacing conventional synthesis methods with more advanced ones which will help to expand the application scope of these ‘green-synthesized’ NPs to several fields. Current synthesis methods lack the attributes for the upscaling of these nanomaterials to an industrial scale of production. As mentioned above, the primary advantage of using sugarcane wastes is their ample availability and low cost which should make them more desirable and sustainable for up-scaled production. There also remains a disconnection with studies pertaining to nanoparticle synthesis and studies demonstrating their application. The majority of current investigations are solely focused on the synthesis without a much-dedicated focus on the application part. Therefore, there is a need for comprehensive research studies with extensive characterisation and a practical model to realise the full potential of sugarcane-derived nanomaterials for a wide range of application fields.

## 3. Sugarcane-derived nanocellulose and related composites

Among different biomasses, sugarcane bagasse has one of the highest cellulose contents. The fibrous pulp contains about 40–50% tissue component as cellulose with another 25–35% being hemicellulose. As a nanomaterial, the synthesis of cellulose nanostructures has been reported in two forms: fibre-like cellulose nanofibers (CNFs) (also called cellulose nanofibrils or nano fibrillated cellulose) and spherical or rod-like cellulose nanocrystals (CNCs) [92]. Among the two nanostructures, CNFs possess both amorphous and crystalline structured cellulose chains with sizes of several microns long while the CNCs are highly crystalline with lengths <500 nm [93]. The cellulose-based nanocrystal and nanofiber materials derived from sugarcane bagasse are frequently reported [94–98] and are discussed in this section covering the applications and different characterisation techniques with a focus on improving their mechanical and thermal properties.

### 3.1. Cellulose nanocrystals (CNCs)

**Figure 7.**
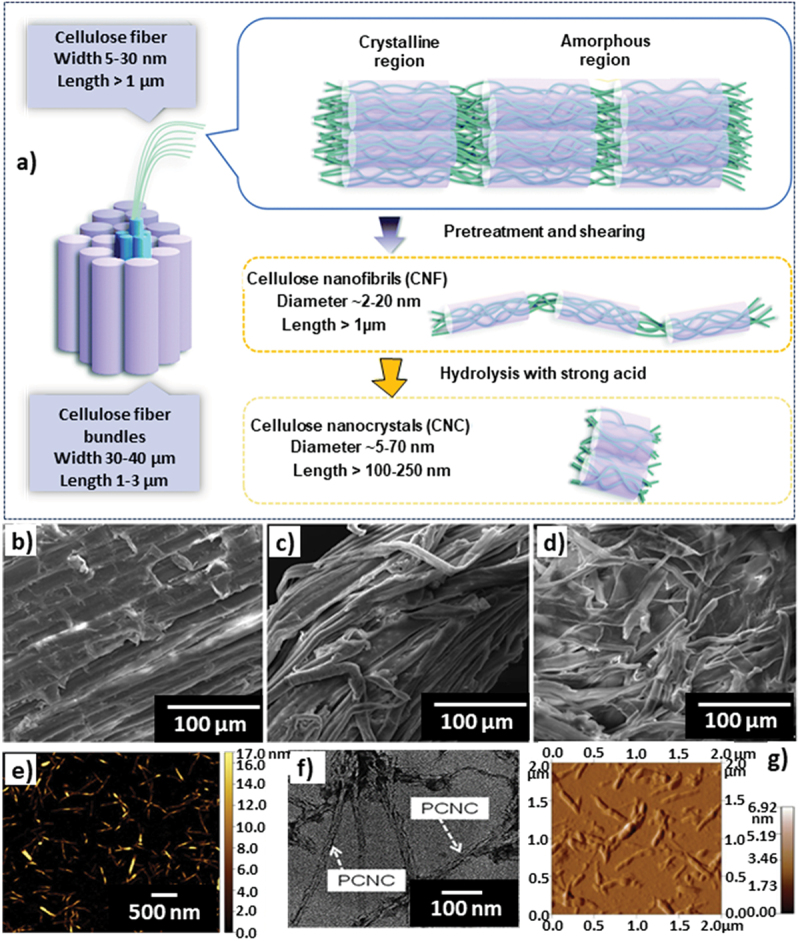
a) Schematic diagram of the synthesis of cellulose nanofibrils and cellulose nanocrystals from bundled cellulose fiber bundles, reproduced with permission [99] copyright 2021, royal society of chemistry, b-e) scanning electron micrographs, left to right: raw SCB; organosolv pretreated SCB and bleached SCB, AFM image of CNC, reproduced with permission [102] copyright 2018, Elsevier, f) TEM image of CNC synthesized using phosphorylation method, reproduced with permission [103] Copyright 2021, Taylor & Francis, g) AFM image of CNC suspension, reproduced with permission [104] copyright 2020, Springer.

The CNCs derived from sugarcane bagasse (CNCs-SB) are generally prepared using three general steps: first, a pre-treatment is done to remove dirt/soluble extracts, which is then followed by bleaching (involving the use of KOH or sodium chlorite) leading to its delignification, and finally an acid/enzyme hydrolysis treatment using a strong acid-like sulphuric acid results in breakage and degradation of the amorphous domains leaving behind the crystalline domains possessing rod/needle-like CNCs of nanoscale dimensions (Figure 7a) [99]. Sulphuric acid hydrolysis is the most common reagent utilized for the preparation of CNCs-SB. Azeez et al. prepared high surface area CNCs-SB by use of sulphuric acid hydrolysis and NaOH treatment and employed these for adsorption of methyl orange (MO). The CNCs-SB showed an almost 3 times better MO adsorption capacity than bagasse by itself [100]. In another similar study, Attia et al. tested sulphuric acid hydrolysed CNCs-SB for the adsorption of MB dye in aqueous systems. CNCs-SB showed a surface area of over 40 times in comparison to purified bagasse lignin, while an improvement of adsorption capacity by factors of 4 and 2.5 compared to bagasse lignin and bagasse carbon fibre, respectively, was observed [101]. Ferreira et al. improvised the conventional CNCs-SB synthesis methods by adding an organosolv pre-treatment using adipic acid before the bleaching step. There is a visible disintegration in the sugarcane bagasse structure as evident from the SEM images (Figure 7b–d). The authors found the particle length decreased from 413 nm to 242 nm, particle width decreased from 10 nm to 6.8 nm and a slight improvement in the material’s crystallinity was also observed. Further, the adipic acid functionalisation resulted in an increase of contact angle from 29° to 38°, thereby reducing its hydrophilicity and enabling dispersion in chloroform [102]. The dispersed CNCs are visualised using AFM, and the images are displayed in Figure 7e. In another work, unlike the traditional sulphuric acid hydrolysis, phosphoric acid (a weak acid) was chosen as a suitable alternative for making CNCs-SB with sodium chlorite as a bleaching agent [103]. The hydrolysis of phosphoric acid creates phosphate groups and further promotes the formation of char, which in turn results in flame inhibition property. Here, the increase in phosphoric acid concentration resulted in higher crystallinity (for 11 M H_3_PO_4_); however, the less concentrated phosphoric acid treatment (5 M H_3_PO_4_) led to the formation of CNCs-SB nanorods with less agglomeration and sizes of approximately 190 nm in length and 4 nm in width [103] as visualised using TEM imaging in Figure 7f.

Making nanocomposites or nanohybrids of CNCs-SB with other materials can effectively increase the surface area of the resultant material while also improving its mechanical properties, thereby enabling potential use in various other applications. Sucharitpong et al. used CNCs-SB to mix with Nylon-6 at different weight ratios to prepare a reinforced Nylon-6 CNC-SB composite. From the different combinations, the 1% wt. ratio provided with a homogenous dispersion, and a large surface area. The morphology of this material was visualised using AFM imaging (Figure 7g) [104]. Leão et al. prepared a composite using CNC-SB with different lengths (150 nm and 220 nm by varying the synthesis procedure) and added acrylonitrile butadiene styrene (ABS). By optimising the weight percentage of CNCs-SB from 0.5% to 1.5%, the tensile strength, tensile modulus, and impact strength were significantly enhanced in comparison to the pure ABS, thus demonstrating the reinforcement effect of CNCs-SB even at a very small amount [105]. In another work, CNC-SB with 200–300 nm in length and 20–40 nm in diameter were used to make composite whey protein isolate to improve the mechanical properties and enhance the hydrophilic nature making it an excellent choice for food packaging [105]. The tensile strength and the tensile modulus were increased by ~2.1 times and ~3.3 times, respectively, when incorporating CNC-SB at an 8% wt. proportion. At the same time, the water solubility was improved by ~1.2 times favoured by the hydrogen bonding from surface abundant −OH groups in CNCs-SB. The mechanical properties of poly(vinyl alcohol) (PVA) can also be improved by making a composite with CNCs-SB [106]. For instance, Pavalaydon et al. prepared CNCs-SB with particle sizes of 48 µm and 347.8 nm for 2 wt% NaOH and 17.5 wt% NaOH treatment conditions, respectively. Notably, the tensile strength and Young’s modulus were improved by a factor of ~1.9 and ~2.3 times, respectively, by the optimised CNCs-SB loading and NaOH pre-treatment concentration compared to the pure polyvinyl alcohol. Pavalaydon et al. demonstrated a similar improvement in the mechanical properties of PVA by making a composite with CNCs-SB [106]. CNCs-SB are also used as a reinforcing agent in tissue engineering scaffolds due to its properties such as biodegradability, biocompatibility, large surface area, and excellent mechanical properties. For instance, PVA/CNC-SB/hydroxyapatite was prepared with a crystallinity index of 59%, a porosity of 58%, and an equilibrium swelling of 532% offering a cell viability of 85% ± 0.92% [107].

**Figure 8.**
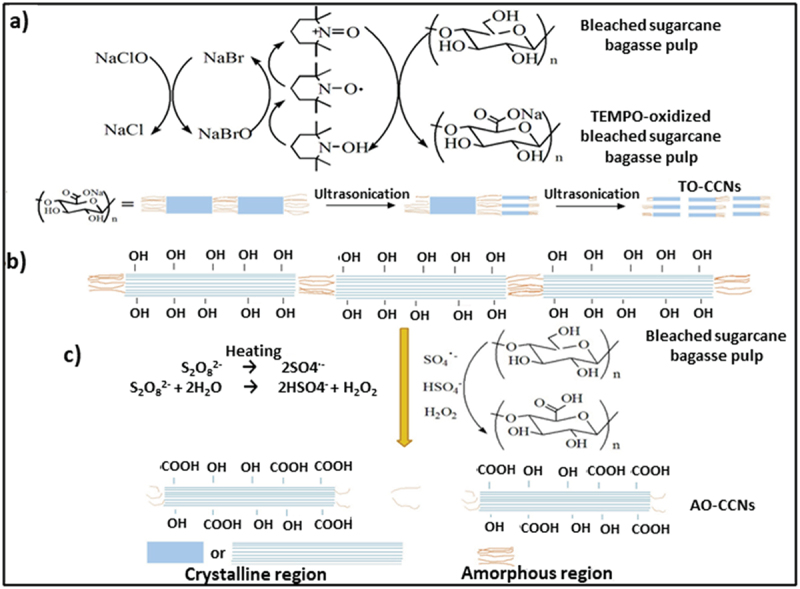
a) Preparation of carboxyl-modified cellulose nanocrystals (CCNs) by two methods, b) preparation of tempo-oxidised CCNs (TO-CCNs), and c) one-step preparation of APS oxidised CCNs (AO-CCNs), reproduced with permission [108] copyright 2016, Elsevier.

Zhang et al. prepared carboxylated CNCs from sugarcane bagasse using 2,2,6,6-tetramethylpiperidinyl-1-oxy radical (TO-CCNs) and ammonium persulfate (AO-CCNs) as oxidants with crystallinity index of 63.3% and 40%, respectively. As expected, both materials exhibited an increase in carboxylate groups, a decrease in yield and a decrease in the degree of polymerization. Notably, TO-CCNs were able to deliver higher carboxyl content when compared to AO-CCN. However, it resulted in comparably less thermal stability than AO-CCN. The AO-CCN showed smaller dimensions as the ammonium persulfate (APS) concentration was increased, while some spherical particles were also observed for the highest APS concentration (Figure 8a–c) [108].

Similar to acid hydrolysis, enzyme hydrolysis is also used for making CNCs-SB with high crystallinity and high cellulose conversion [109,110]. Using semi-solid fermentation of fungus *Aspergillus fumigatus* CCT 7873, enzymatic hydrolysis was carried out using NaOH and Ca(OH)_2_ pre-treatment [110]. On increasing the concentration of Ca(OH)_2_, the cellulose percentage can be increased with a crystallinity index as high as 50.78% for 20% Ca(OH)_2_ pre-treatment. At the same time, even with 4% NaOH pre-treatment, 56.63% of cellulose and 52.99% crystallinity index could be obtained which is higher compared to that of the samples prepared with Ca(OH)_2_ pre-treatment. Steam explosion and liquid hot water are two pre-treatment methods for the preparation of CNCs. In steam explosion, the application of high-pressure steam for a short duration is done followed by rapid release of pressure enabling the formation of CNCs. In the case of liquid hot water treatment, using hot water as solvent at high temperature, the cellulose structure is broken to form CNCs. Using these pre-treatment strategies with the combination of enzymatic and sulphuric acid hydrolysis, a high crystallinity index of 81% is obtained with a length/diameter aspect ratio of 11–15 with the second-generation ethanol process using sugarcane bagasse as precursors [109].

### 3.2. Cellulose nanofibers (CNFs)

The sugarcane bagasse-derived cellulose nanofibers (CNFSBs) are mainly synthesized through various mechanical methods including high-frequency ultrasonication, high-pressure homogenisation, ball milling, and micro-fluidization [53]. Although the mechanical methods require a lot of energy, these are more environmentally friendly considering the reduced use of chemicals.

High-pressure homogenization is the most common mechanical method to produce CNFs using facile methodology by the application of high pressure, velocity, and shear to denature the amorphous regions of bagasse. The resultant product is a nanomaterial possessing a high surface area and a high aspect ratio [111]. High-pressure homogenization and ultrafine grinding techniques were employed by Lu et al. to obtain 20 nm width CNFs from bleached bagasse pulp. The results of these studies showed that a lower concentration of hemicellulose content led to a higher crystallinity index and thermal stability, whereas a higher concentration of hemicellulose content increased the activation energy [112]. In another report, Nie et al. utilized xylanase and NaOH pre-treatment on unbleached SB pulp followed by grinding and high-pressure homogenization to prepare CNFSB [113]. Here, after the xylanase pre-treatment, a crystallinity index of 66.6% with typical cellulose I structure was obtained, whereas on increasing NaOH concentration, a gradual formation of cellulose II structure is observed evidently through the XRD peaks at 11.5° and 20.5° consequently improving the thermal stability as well. These were shown to further improve the crystallinity index along with improvement in the thermal stability of the compound [114]. Lan et al. produced CNFSB using p-toluenesulfonic acid pre-treated bagasse with a secondary treatment of formic acid or HCl followed by high-pressure homogenization. Comparing the two secondary treatments, the HCl-treated CNFs were found to be more crystalline than the formic acid-treated samples; however, the latter possessed a significantly smaller average particle size and a larger contact angle contributing to an improved dispersity [115].

Ultrasonication is often used for fibrillating the biomass through ultrasonic waves as a way to increase the cellulose content and to improve its mechanical properties. Somvanshi et al. prepared PVA/CNFSB using an ultrasonication technique and tested it for their mechanical, thermal, and antimicrobial properties [116]. Notably, the reinforced PVA/CNFSB sample prepared with 40% CNFSB, acetic acid and NaOH treatment along with sonication outperformed other samples delivering large tensile strength, large elongation percentage, and a large elasticity modulus. At the same time, when checked for antimicrobial properties, the PVA reinforced with CNFSB film exhibited an inhibitory zone diameter of 13.42 mm and 15.21 mm for *E. coli* and *S. aureus*, demonstrating its potential application in food packing for enhanced shelf life [116].

To obtain a high aspect ratio, uniform size, and high mechanical properties of CNFSB, three-step mechanical force treatment involving grinding, high-pressure homogenization, and ultrasonication was performed by Zhang et al. The materials obtained using this strategy possessed an average diameter as small as 23.18 nm, along with high crystallinity, while the films prepared showed high tensile strength and high strain at break [117]. Feng et al. prepared high aspect ratio and 20–30 nm ranged CNFSB using a series of chemicals (steam explosion, NaOH catalysed hydrothermal treatment and H_2_O_2_ bleaching) and mechanical treatment (high-speed blending, ultrasonication) [118]. Notably, the crystallinity of SCB increased from 55.1% to 73.6% after chemical treatment, whereas it was lower in the case of the mechanical method of synthesis. The CNFSB also exhibited high thermal stability with the increase of T_on_ from 260.4° (SCB) to 301.1° (CNFSB).

Using pre-treatment prior to the mechanical treatment can result in increased yield and reduced energy consumption. For instance, enzyme pre-treatment (using mono-component endoglucanase) carried out before ultrafine grinding recorded an enhanced yield of 30.57% and reduced energy consumption by 59.71% while offering more uniform CNFSB and reducing the diameter by ~2.7 times [119]. Moreover, the transparency of the CNFSB film with enzyme treatment showed a transmittance of 60.02% which is higher compared with the sample without enzyme pre-treatment. In another study, Liu et al. prepared CNFSB by the variation of endoglucanase pre-treatment (0–120 IU/g) and mechanical grinding and were able to tune the length (298–4500 nm), diameter (9.1–26.3 nm), aspect ratio (25.3–192.3) and crystallinity (51–75.1%) of the nanomaterial while reducing the energy consumption [120].

While the dosage of endoglucanase was increased, transmittance and the elastic modulus were enhanced but the tensile strength and the elongation at break decreased significantly, thus exhibiting excellent mechanical properties for all the prepared materials. Enzymatic pre-treatment using cellulase was done on SB to produce CNFSB using grinding and high-pressure homogenization [121]. Notably, the cellulose pre-treatment could increase the crystallinity of SB from 56% to 63% while increasing the thermal stability.

Luo et al. prepared CNFSB by varying the pre-treatment process such as hot water, green liquor, and sodium chlorite with crystallinity index of 54.7%, 61.4%, and 67.1%, respectively, [122]. Notably, the CNFSB from hot water and green liquor pre-treatment exhibited almost 100% UV resistance, whereas the CNFSB from sodium chlorite possessed a very low lignin content (1.8%), resulting in a low UV adsorption rate. 2,2,6,6-tetramethylpiperidine-1-oxyl (TEMPO) mediated oxidation is an acid-free pre-treatment strategy capable of developing materials with excellent mechanical properties while reducing the energy requirements of the mechanical methods. Using TEMPO-mediated oxidation, CNFSB (length 150 nm to 600 nm, diameter 1 nm to 5 nm) were synthesized and further made into a composite with PVA with the CNFSB weight percentage from 10% to 50% [123]. The addition of CNFSB is highly effective in reducing the solubility of PVA in water from 58.9% (PVA) to 39.1% (50% CNFSB/PVA) while a decreasing trend in transmittance is observed with an increase in CNFSB content. Moreover, the 30% CNFSB/PVA registered the highest tensile strength of 6.6 kPa, whereas the increase in CNFSB was found to increase the swelling characteristic of the sample consistently.

**Figure 9.**
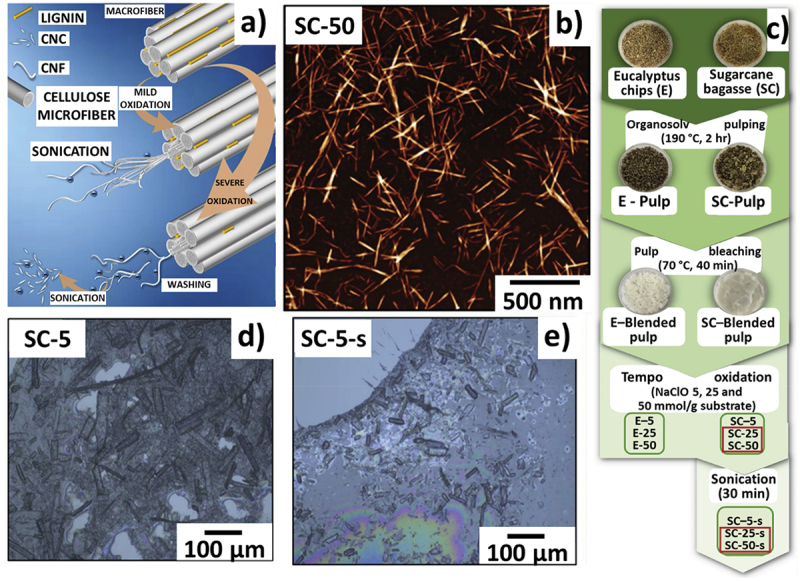
a) Schematic representation of CNC and CNF with the effect of a mild and high degree of oxidation b) AFM image of CNF c) synthesis protocol of CNF and CNC with real photographic images, and d, e) optical image of CNF and CNC, reproduced with permission [127] copyright 2019, Elsevier.

**Figure 10.**
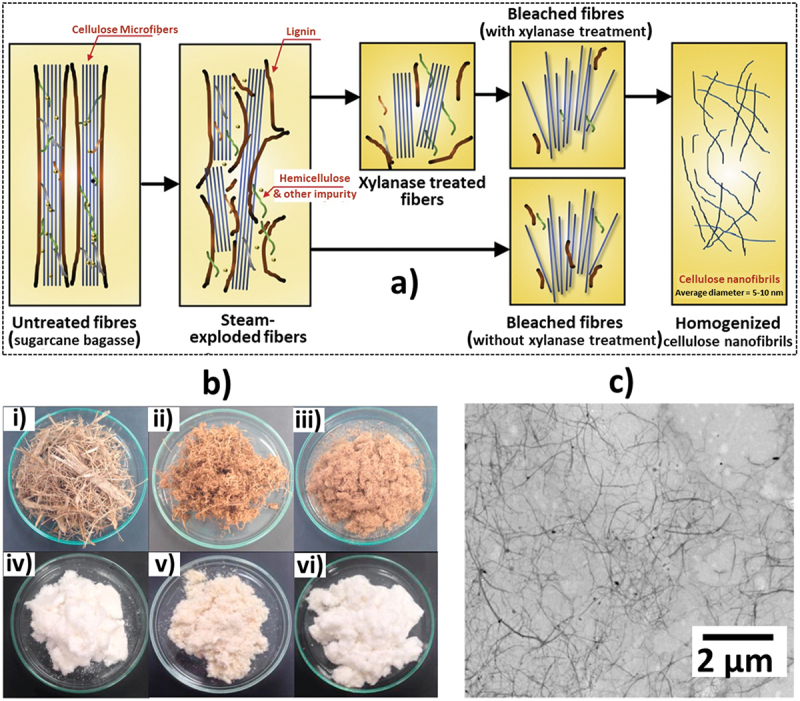
a) Schematic illustration of homogenised CNF preparation using xylanase treatment b) photographs of (i) SCB (ii) SE-SCB steam exploded (iii) SE-SCB-X20 steam exploded, 20 U/g xylanase treatment (iv) SE-SCB-X20-B steam exploded, 20 U/g xylanase treatment, bleached 5 times (v) SE-SCB-B steam exploded, 5 times bleached (vi) SE-SCB-B steam exploded, 9 times bleached, and c) TEM of CNF, reproduced with permission [111] copyright 2016, Elsevier.

Chinga-Carrasco et al. prepared CNFSB by soda pulping combined with a hydrothermal treatment followed by TEMPO oxidation [124]. The noncytotoxic and highly viscous nature of these materials enabled their use in 3D printing as inks with high resolution and stability. Syrový et al. prepared CNFSB and CNFSB/PEG (poly(ethylene glycol)) using a hydrothermal treatment and soda delignification combined with TEMPO oxidation [125]. Interestingly, when used as a humidity sensor, a response time of 265 s is achieved for CNFSB/PEG and 200 s for CNFSB, whereas CNFSB/PEG (490 s) outperformed CNFSB (1020 s) in terms of recovery time. Using NaOH pre-treatment and oxidation (using TEMPO and NaClO), CNFSBs were prepared [126]. As expected, the increase in concentration of NaClO resulted in enhanced oxidation of the amorphous regions forming smaller dimensional CNFSB and increased crystallinity index (32% to 61%). Pinto et al. prepared CNF and CNC from SCB using organosolv pulping, pulp bleaching and TEMPO oxidation (Figure 9a,c). Here, the high degree of oxidation resulted in 3 to 5 nm sized CNFs as evident in AFM (Figure 9b). The effect of 30-minute sonication resulted in reduced turbidity of dispersion in the samples to form CNC (Figure 9d,e) [127]. Steam explosion is a facile and low-cost environmentally friendly pre-treatment which uses high-pressure steam from water for the removal of unwanted impurities and effective removal of non-cellulosic content of biomass [128]. A multi-step approach including steam-explosion pre-treatment (by varying temperature and duration), NaOH pre-treatment, hydrogen peroxide bleaching, high-speed agitation and high-pressure homogenization method was carried out to prepare CNFSB [128]. The prepared nano-fibrillated samples exhibited significantly increased cellulose content resulting in a diameter range of 3 to 7 nm, low density (0.8989 g/cm^3^) and moisture content of 6.3264 ± 0.3352 wt%. Saeleea et al. used steam explosion and xylanase pre-treatment followed by sodium chlorite bleaching and high-pressure homogenization at 15,000 psi/30 passes to obtain CNFSB with <10 nm diameter and less agglomeration (as in the TEM image, Figure 10a–c) [111]. Figures 10a,b show the schematic preparation of the samples and the photographs of samples at different stages of preparation (steam explosion, xylanase treatment, bleaching and high-pressure homogenisation). The results showed that the high-pressure homogenization process deformed some of the crystalline regions resulting in a decrease of crystallinity (from 69.7% to 68.1%) and thermal stability (T_on_ from 300 °C to 270 °C) compared to the bleached fibre without the mechanical treatment.

Ionic liquids have low vapour pressure and non-flammability making it an environmentally friendly and highly efficient treatment method for developing high thermal and chemically stable materials [129]. Sankhla et al. prepared CNFSB from cellulose micro-fibers by nano-fibrillation technique using 1-butyl-3-methylimidazolium chloride as ionic liquid where Na_2_CO_3_ is used for pre-treatment (for micro-fibrillation) instead of the commonly used strong alkaline NaOH [129]. Notably, the yield of nano-fibrillation was 85% and the sizes of the achieved CNFSBs are an average diameter of 18 nm and a few micrometres ranged length.

Making nanocomposites or nanohybrids or doping can alter the morphology, surface area and chemical structure of nanostructures, resulting in improved thermal and mechanical properties [130–132]. Sankararamakrishnan et al. prepared Fe(0)@FeS decorated on CNFSB, where the large surface area of CNFSB enables the uniform dispersion of NPs and effectively inhibits agglomeration. These materials were used for the removal of MB and Congo red dye in an aqueous system and demonstrated large adsorption capacity (200.0 and 111.1 mg/g) with only 1.3% and 0.9% reduction in dye removal after 5 cycles showing excellent recyclability [133]. Nie et al. used a vacuum-assisted filtration process for the preparation of CNFSB/aluminium nitride (AlN) films showing excellent thermal and mechanical properties. With the increase in AlN content from 0%, 2%, 5%, 10%, and 20%, the light transmittance achieved for the nanocomposites was 80%, 64%, 35%, 5%, and 0%, respectively, whereas the tensile strength and elongation decreased with the increase in AlN content [114].

Sofla et al. compared CNFSB and CNCSB prepared using ball milling and sulphuric acid hydrolysis and studied their thermal properties, morphology, and chemical structure [134]. The sulphuric acid hydrolysis resulted in a small diameter (20–30 nm) and aspect ratio (11) with high crystallinity (73%) while the ball milling resulted in a comparable larger diameter (50 nm) and high aspect ratio (40) with less crystallinity (68%) which can be related to the effective breaking of the amorphous region by sulphuric acid hydrolysis. In terms of thermal stability, CNCSB was found to be less thermally stable, which is attributed to the sulphuric acid-induced removal of hydroxyl groups and decomposition of CNCSB through catalysis or esterification mechanisms [134].

In summary, both CNFSB and CNCSB are excellent candidates for delivering excellent mechanical and thermal properties with rod/needle-shaped particles in nanometre dimension. Various chemical and mechanical synthesis methodologies were reported with high yield, high surface area and high crystallinity; especially, their mechanical properties were extensively studied. However, only a few works on hybrids, composites or doping have been studied to date. Hence, further research focus is needed. Considering their low cost, environmental friendliness and renewability, these materials have a high potential for commercialisation through innovative techniques for reducing energy consumption and reducing the usage of harmful acids. Further, research on extending the materials prepared to new applications is also recommended. A summary of various aspects of nanocellulose-based materials is provided in Table 2.

**Table 2.** Summary of synthesis methods, properties and applications of cellulose nanomaterials derived from sugarcane-based products.

Material	Method	Property/Application	Ref.
Cellulose nanocrystals (CNCs)	Sulphuric acid hydrolysis and NaOH treatment	Dye adsorption	[100]
	Sulphuric acid hydrolysis	Dye adsorption	[101]
	Organosolv pre-treatment using adipic acid	Novel Synthesis	[102]
	phosphoric acid hydrolysis and sodium chlorite treatment	flame inhibition properties	[103]
	Composite with Nylon-6	Improved mechanical properties	[104]
	Hybridization with acrylonitrile butadiene styrene (ABS)	Improved tensile strength	[105]
	Composite with whey protein	Food packaging	[105]
	Composite with of poly(vinyl alcohol) (PVA)	Improved mechanical properties	[107]
	Carboxylation with tetramethylpiperidinyl-1-oxy radical (TO-CCNs) and ammonium persulfate (AO-CCNs)	Decrease in degree of polymerisation	[108]
	Steam explosion	Novel synthesis	[109]
	Enzyme hydrolysis	Increased cellulose content	[110]
Cellulose nanofibers (CNFs)	High-pressure homogenization	High thermal stability	[112]
	NaOH pre-treatment and high-pressure homogenization	High thermal stability	[113]
	p-toluenesulfonic acid pre-treatment and HCl/Formic acid secondary treatment	High crystallinity/smaller particle size and dispersity	[115]
	PVA/CNFSB and ultrasonication	Anti-microbial properties	[116]
	Three-step synthesis: grinding, high-pressure homogenization, and ultrasonication	Improved mechanical properties	[117]
	Chemical and mechanical treatment combinations	Improved mechanical properties	[118]
	Enzymatic pre-treatment	Improved thermal stability	[121]
	Pre-treatment with hot water, green liquor, and sodium chlorite	UV resistance	[122]
	TEMPO-mediated oxidation	Increased tensile strength	[123]
	Hydrothermal treatment and TEMPO-mediated oxidation	3D printing ink application	[124]
	Hydrothermal treatment and soda delignification combined with TEMPO oxidation	Novel synthesis	[125]
	Organosolv pulping, pulp bleaching, and TEMPO oxidation	Novel synthesis	[127]
	Steam-explosion pre-treatment	Increased cellulose content	[128]
	Steam-explosion and Xylanase pre-treatment	Improved thermal stability	[111]
	Doping with Fe(0)@FeS	Dye adsorption	[133]
	Doping with aluminium nitride (AlN)	Improved mechanical properties	[114]
	Ball milling and sulphuric acid hydrolysis	Improved thermal stability	[134]

## 4. Sugarcane-derived nano biochar

**Figure 11.**
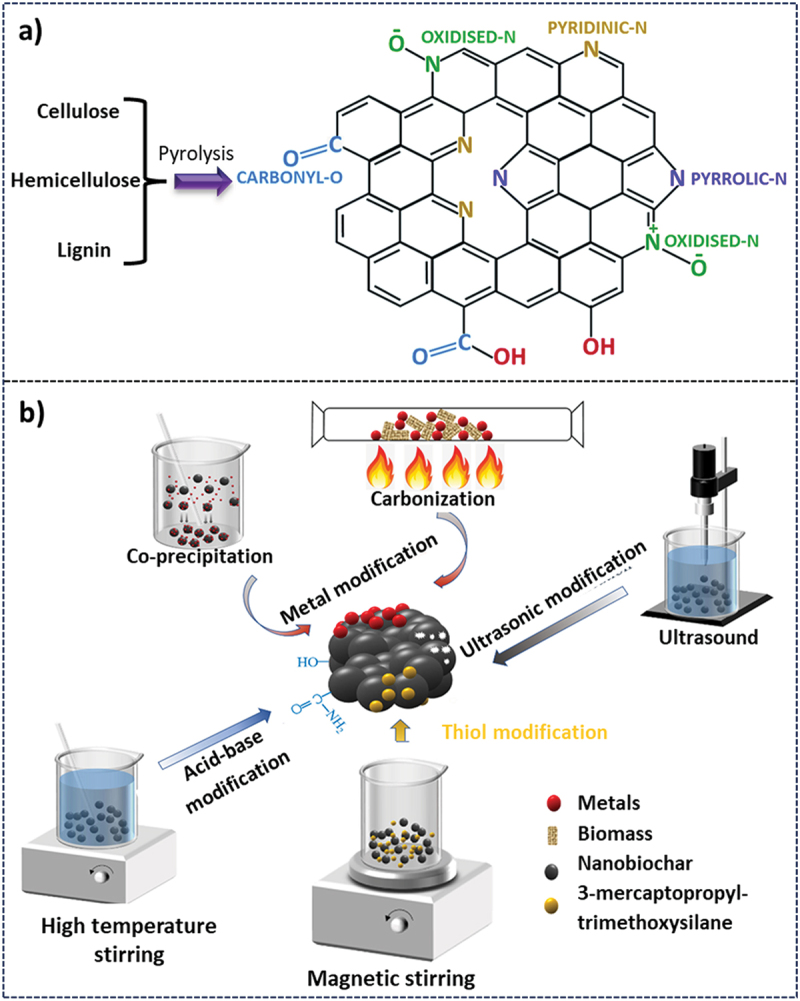
a) The molecular structure of biochar, reproduced with permission [14] copyright 2017, royal society of chemistry, and b) synthesis and modification of nanobiochar using different methods described in the literature, reproduced with permission [145] copyright 2023, Springer.

Biochar is a charcoal-like material that is produced from biomass under high-temperature, oxygen-free/oxygen-limited conditions [135,136]. Based on the nature of the precursor and the synthesis conditions, biochar can be tailored to possess different physico-chemical properties including their elemental composition, surface area, porous nature, and functional groups [137]. Structure-wise, biochar is a condensed network of aromatic carbon atoms with several types of surface functional groups attached at the terminal ends produced during high-temperature treatment. The pyrolysis temperature and the residence time are the main controlling factors that can determine the overall distribution of the carbon network in five or six-membered rings in biochar and the type of surface functional groups, which may include carbonyl (C=O), carboxylic (COOH), oxidised-N, pyrrolic-N, and pyridinic-N (Figure 11a) [14]. The surface functional groups in biochar are also dependent upon the composition of biomass in terms of the three biopolymers including cellulose, hemicellulose and lignin. Biochars are extremely relevant to the sugarcane agroindustry as several of its waste and by-products are ideal feedstocks for producing biochars, two of the most prominent being bagasse and trash waste [138]. In this regard, bagasse-derived biochars are generally produced under high temperature/pressure conditions to maximise the purification of carbon and crystallization of silica present in the straws. These are generally highly porous and have a high surface area [139]. Contrastingly, trash waste is a rich pool of secondary elements and micronutrients. To preserve these in their bioavailable form, trash biochars are synthesized at relatively lower temperatures partly compromising on the quality of the charred carbon. While their surface area is lower than that of bagasse-derived biochars, they contain greater amounts of nutrients, surface functional groups and exchangeable cations [139]. Properties of both these types of biochars can be enhanced through the process of activation and by their functionalisation with other chemical agents [17].

A smaller particle size is desirable in biochar as the greater surface-to-volume ratio provides for higher adsorption capacity. Biochars having particle size in the nanoscale (<100 nm) have been termed ‘nanobiochars’ [140]. Nanobiochars with their superior thermochemical stability and greater number of functional groups offer potential applications in several fields and have gained considerable research interest as a useful nanomaterial [141,142]. Their use as soil amendments and as contaminant sorbents have been well demonstrated in several pieces of literature along with their uses in several other fields including energy storage, biofuels, catalysis, and material science [143].

Nanoscale biochar particles can be obtained either by making use of special pyrolysis conditions, by adding a subsequent exfoliation step or by a combination of both. Today, the most common technique for nanobiochar synthesis involves a mechanical breakdown of biochar particles using a ball-mill grinding [143]. In their study, Lyu et al. showed how the ball milling technique showed a dramatic increase in the surface area of bagasse and other agro-waste-derived biochar. In the case of bagasse-biochar pyrolysed at 450 °C, ball milling enhanced the surface area by a remarkable 6-fold [144]. Ball milling also showed improvement in other surface properties of biochar including pore volume and oxygen functional groups and promoted greater electrostatic interactions [144]. Apart from these, other synthesis techniques including flash pyrolysis, acid digestion and ultrasonication have also been studied for the production of nanobiochars (Figure 11b) [145]. While nanobiochars are extremely versatile compounds by themselves, hybridizing them with other nanomaterials in the form of nanocomposites has provided further specialized properties [146]. A majority of these nanocomposites have been used in environmental applications, especially as adsorbents for effective heavy metal and organic matter treatment from wastewater. High surface area, porosity, and presence of polar functional groups such as carboxylic, hydroxyl, and amino groups found on biochar surfaces add to their capabilities as effective sorbents. Simultaneous adsorption and catalytic degradation of contaminants is a property unique to nanobiochar composites. Bagasse-biochar nanocomposites have been widely explored to function as chemical adsorbents [146].

Zhang et al. in their work produced a MgO-sugarcane bagasse biochar nanocomposite amongst others including MgO composites with sugar beet tailings, pine woods, cottonwoods, and peanut shell biochars [147]. These nanomaterials were tested for their efficacy in adsorbing nitrates and phosphates from aqueous solutions, a major cause of eutrophication in water bodies. The study showed bagasse nanobiochar composites could successfully be used as a sorbent for phosphate and nitrate contaminants in wastewater samples. Zhang et al. in a previous work had also demonstrated the potential use of a bagasse biochar-based ferromagnetic composite for arsenic removal for wastewater treatment [148]. A similar MgO-biochar-based composite was also prepared using sugarcane leafy trash for the adsorption of heavy metal pollutants including arsenic (As (V)), lead (Pb (II)), and MB dye from water. The highest adsorption was noted for MB dye followed by As (V) and Pb (II) [149]. The effect of increasing temperature and ionic strength was negatively proportional to the composite’s adsorption capability.

**Figure 12.**
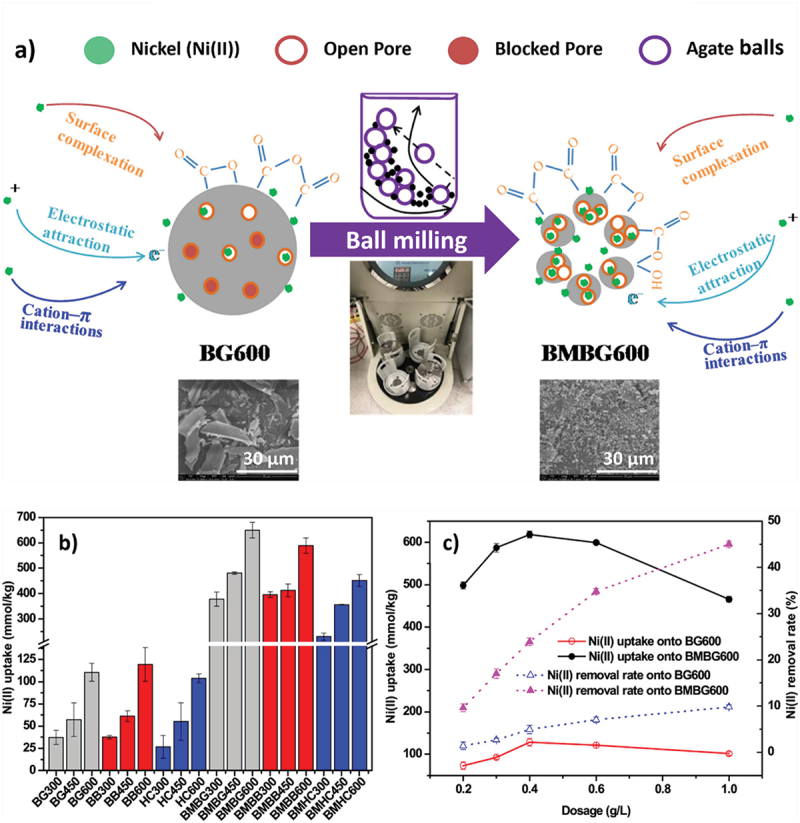
a) Mechanism of Ni (II) adsorption on bagasse biochar pyrolysed at 600 °C (BG600) and ball-milled nanobiochar (BMBG600) b) effect of feedstocks and temperature on Ni (II) adsorption, and c) dosage on Ni(II) removal efficiencies from aqueous solution by unmilled and milled biochars, reproduced with permission [152] copyright 2018, Elsevier.

In another study, Gan et al. produced a Zn-bagasse biochar nanocomposite for the removal of chromium (Cr (VI)) from contaminated aqueous solutions. Cr (VI) is an extremely hazardous heavy metal contamination that can potentially cause bioaccumulation in human populations, and hence, its removal is of prime priority. To synthesize the composite, a mixture of Zn precursor and bagasse powder was pyrolyzed at 450 °C to form a highly porous nanobiochar composite. It was further characterized that this composite performed the best in acidic conditions and could also be reused following a pollution treatment adding a unique recyclable quality to the material [150]. Another manner in which biochar-derived nanocomposites are prepared is by coating material on the biochar surface. Zhou et al. successfully demonstrated chitosan coating on bagasse-derived biochar for the purpose of heavy metal adsorption [151]. Currently, nanobiochars are most commonly used for adsorption of pollutants, including heavy metals and dyes. In two of their works, Lyu et al. demonstrate the application of sugarcane-bagasse-and-other agro-waste-derived nanobiochars in the adsorption of pollutants including nickel (II) and MB dye due to their enhanced physico-chemical properties [144,152]. The synthesis, modifications and adsorption studies of their work are shown in Figure 12. Nanobiochar and its derived nanocomposites also find a variety of uses in distinct industries and processes including in catalysis, energy storage, supercapacitors, and biomedical sensors [153]. Lately, nanobiochars are also being used as fuel additives to mitigate the emissions of toxic greenhouse gases. Ardebili et al. demonstrated the use of sugarcane bagasse-derived nanobiochar as a fuel additive. Different concentrations of sugarcane bagasse-based nano biochar were added to the diesel fuel leading to a significant improvement in engine performance metrics along with a reduction in exhaust emissions including, NO_x_, UHC, and CO emissions to up to 20%, 25% and 33%, respectively [154].

## 5. Specific application perspectives

As mentioned earlier, the SB-derived nanomaterials, including biocarbon, CNC/CNF, and silica, have exhibited initial but promising potential, particularly following their hybridisations with other materials allowing for the development of unique physicochemical properties and their application into various fields. SB-derived nanomaterials have been employed to fabricate electrode materials for batteries owing to their attractive features such as surface-active sites, large surface area, high wettability, increased conductivity of hybrid composites, and large pore volume to accommodate expansion issues during cycling. For instance, Praneetha and Murugan developed a three-step microwave-assisted method to synthesize porous Si with carbon nanohybrids for LIB application [155]. Other than showing significant electrochemical performance, the SB-derived Si owned a highly crystalline internal nanoporous structure that could behave as the buffer nanospace and accommodate the volume change, but the commercial Si electrode film suffered from cracking and electrical disconnection. Zhu’s group successfully loaded β-FeOOH on SB-derived functionalized porous carbon (FPC) as an anode for LIB [156]. The as-prepared FPC/β-FeOOH nanocomposite presented a hierarchically porous structure with a high specific surface area of 1443.7 m^2^ g^−1^ and showed a high discharge capacity of 898.8 mAh g^−1^ after 350 cycles at 0.2 A g^−1^, compared to a bare FPC of 465.7 mAh g^−1^ and the charge transfer resistance was accordingly decreased from 255 Ω to 70 Ω after coupling with FeOOH.

Apart from energy storage, SB-derived nanomaterials have also demonstrated potential for adsorption. Different techniques have been developed in recent decades to treat contaminated effluents by utilizing SB-derived nanomaterials. Pehlivan et al. obtained polysaccharides from SB through the hydrolysis method in an acid/alkaline solution and then mixed the treated SB with Fe(NO_3_)_3_ to remove As(V) in aqueous solutions [157]. The prepared SB/hydrated ferric oxide (SB/HFO) composite contains a large number of carboxyl and hydroxyl groups, which could be substituted by negatively charged As(V) anions like H_2_AsO^4-^ and HAsO_4_^2-^ due to electrostatic force, and the positively charged ≡FeOH_2_^+^ possibly chelated with As(V) anions under acid condition, resulting in effective adsorption. Therefore, SCB-HFO exhibited the adsorption capacity of 22.1 mg g^−1^ at pH 4 for As(V), which was able to be reversibly desorbed under 1 M NaOH solution. Zhao and colleagues developed a new type of composite composed of ultrafine Fe_3_O_4_ NPs and SB-derived porous graphitic carbon nanosheets (PGCN), which was realized via a two-step pyrolysis and incipient-wetness impregnation treatment method [158]. The as-proposed Fe_3_O_4_/PGCN nanocomposite exhibits a hierarchical porous structure, with a specific surface area and total pore volume of 1692.6 m^2^ g^−1^ and 1.05 cm^3^ g^−1^, respectively. The good surface wettability and negatively charged property of the Fe_3_O_4_/PGCN nanocomposite, together with its high surface area and conductivity, enable its excellent capacitive deionization (CDI) performance as a cathode combined with a commercial activated carbon (A-AC) anode to remove heavy metal ions of drinking water. The A-AC//Fe_3_O_4_/PGCN HCDI system’s removal capacities were 20.9 and 20.2 mg g^−1^ toward dilute 20 mg L^−1^ Pb^2+^ and Cd^2+^ in water, with efficiency larger than 95% at pH ranging from 5.5 to 6.5. Excellent regeneration and recycling durability was also achieved in a mixture of multiple heavy metal ion solution with a concentration of 0.5 mg L^−1^, where more than 90% of every heavy metal ion (Mg^2+^, Zn^2+^, Cu^2+^, Cd^2+^, and Pb^2+^) has been successfully removed. Overall, SB-based nanomaterials have shown immense potential for specific applications of energy storage and conversion and removal of dyes; however, it can be expanded into other areas after hybridization with other suitable materials.

## 6. Conclusions and future outlook

The review presented the latest knowledge in the field of nanomaterials derived from sugarcane-based waste products. Sugarcane products have extensively been utilized in several conventional area areas with the most prominent being as a source of energy. Much of these products can also end in landfills leading to environmental concerns. Using these products as a starting precursor for the production of low-cost nanomaterials that could be used for a vast number of applications is a highly sought-after proposition for the conversion of waste to wealth.

In terms of nanomaterials, the highest significance in the published literature is associated with silica NPs as almost all parts of the sugarcane plant are enriched in silica during their growth period. Ashing and chemical treatment of the sugarcane products is a good ploy to extract SNPs, which can then be suitably manipulated in terms of size and shape to suit specific applications such as catalysis or drug delivery. Carbon-based nanomaterials such as carbon dots are another extensively researched area based on sugarcane products. Sugarcane juice is an ideal precursor for this purpose, which can be converted into CDs by treatment with chemicals such as ethanol and by applying high centrifugal force. Sugarcane juice, being rich in sucrose, has also received great attention for controlling the agglomeration of the metallic NPs in the matrix. In such cases, juice can act as a capping agent to prevent the association of NPs, which leads to their increased application efficiency. Copper, zinc and silver-based NPs are some of the prominent materials studied using sugarcane-based products. Sugarcane waste is also a great source of cellulose-based nanomaterials including cellulose nanocrystals and cellulose nanofibers. A chemical method such as acid-based hydrolysis is the most commonly utilised method to convert sugarcane products into cellulose nanocrystals, whereas some other less prominent techniques such as enzymatic hydrolysis can also be applied for the same purpose. Sugarcane products, being rich in carbon in the form of sugars, have also been a hot subject for their conversion into nanobiochar. Such products show high porosity which can be further enhanced using methods such as activation. It is also important to highlight how sugarcane is more useful than other biomass types for the overall utilization to produce nanomaterials. In comparison to other biomass-based products, sugarcane-based waste products are much more appealing to produce nanomaterials, which can be attributed to their low cost; large scale and abundant availability; high silica levels; high fibrous content for nanocellulose production; and a variety of waste products including molasses, bagasse, juice, and green trash waste. These features make sugarcane a versatile plant crop for harvesting a plentiful of waste products for their conversion into useful nanomaterials for various purposes.

**Figure 13.**
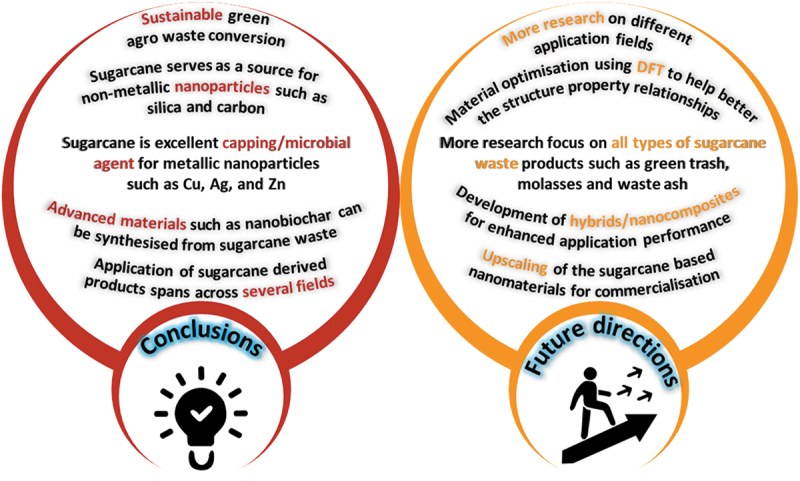
Information on the conclusion and future directions of the review.

An overview of the conclusions and the future outlook is presented in Figure 13. Future research on sugarcane-derived biomass must focus on the following points

1. Improvement in the synthesis procedures; developing novel, hybrid materials; and demonstrating their successful applications in different sectors could be one of the main future research objectives. The new discussion read as “The conventional methods of developing nanomaterials from biomass include high-temperature carbonization, hydrothermal carbonization and other related procedures which can lead to the formation of materials with uncontrolled structure and morphology”. The innovation in the synthesis procedure can be brought about in terms of controlling the structure of the carbonized material via manipulating the experimental parameters and adopting new types of procedures. For instance, the physical mixing of biochar with chemicals can produce better hybrid material properties than the liquid or wet impregnation approach. Biomass itself can be stripped into individual components such as cellulose, hemicellulose and lignin to bring in more homogeneity in the structure of produced carbon products, which can be further chemically or physically activated and modified to produce desired hybrid structures. Microwave-assisted pyrolysis can be combined with a catalyst to speed up the process and at the same time produce materials with exciting properties in a short time frame. Although reported occasionally, the carbonization of biomass can be drawn into innovation by introducing sequential steps to decompose each component of the biomass. This can produce materials with fewer defects and disorders and better physico-chemical properties.
2. What makes these materials attractive is their economic and environmental sustainability. While this has been shown on smaller laboratory scales, processes must be further improved upon and scaled up to actualise these theoretical gains. While utilizing biowaste as a source precursor for producing nanoparticles is undoubtedly an attractive proposition from the cost point of view and the physico-chemical properties of the obtained nanomaterials, their performance relative to the other nanomaterials synthesized using synthetic chemicals/precursors is an active area of research improvement.
3. Efforts must also focus on making hybrid materials to tap the cumulative benefits of two or more constituent materials. These can also be further customised to suit various target industries. Secondly, more efforts now should be directed toward their practical applications. As demonstrated by small-scale experiments, such materials may find extensive use in several industries including materials, environment, energy, biomedicine, and agriculture.
4. Most of the literature on their applications lacks comprehensive studies and therefore only provides limited guidance. Further work is therefore required to fill in the present gaps.
5. The geographic location of the sugarcane biomass and the related waste products could also be given due consideration when looking at the properties of various nanomaterials synthesized from them. In general, it should not make too big a difference; however, considering that different locations may have different growing conditions and accumulation of various elements during the growth of the biomass, it may affect the properties of nanomaterials.
6. The production cost of producing biochar from biomass is a culmination of a range of factors including procurement of the source biomass, transport, storage, carbonization and further distribution of the final product. Considering that carbonization or pyrolysis is the main step involved in the conversion of biomass to useful products, we analyzed the available literature to ascertain the related expenses. While it is hard to locate enough literature that directly analyses the electricity price for carbonization, the practical evaluation in terms of the market price of produced carbon has appeared in the literature on numerous occasions; for instance, the minimum carbon price of biochar and fuels produced during slow pyrolysis was estimated to be $642.40 for every tonne [159]. This is a remarkably low cost when compared to other nanomaterials that are produced using synthetic chemicals. Another report highlights the market price of biochar to be £222 per tonne, which could be further reduced if the associated heat and electricity are sold externally [160]. A very recent study in 2024 estimated that 1.94 to 2.67 KJ/Kg K is the average amount of electricity-based heat energy required to carbonize biomass with a 25-35% yield of biochar [161]. The fast pyrolysis of biochar for the production of transportation fuels has been projected to cost ~$0.054/kWh for electricity [162]. Overall, little attention has been paid to the cost analysis for the carbonization of biomass, which is a crucial aspect that needs to be looked upon in the future.

## Abbreviations

nm: nanometer
3D: three dimensional
GO: Graphene oxide
SB: Sugarcane Bagasse
SL: Sugarcane Leaves
NPs: Nanoparticles
SEM: Scanning electron microscope
TEM: Transmission electron microscope
SE: Steam exploded
GNS: Graphene nanosheets
FPC: Functionalized porous carbon
HFO: Hydrated Ferric Oxide
PGCN: Porous graphitic carbon nanosheets
CDI: Capacitive deionization
AC: Activated carbon
CEC: Cation exchange capacity
CD: Carbon dots
CQD: Carbon Quantum dots
SNPs: Silica nanoparticles
BSNPs: Biogenic silica nanoparticles
SBSNs: Sugarcane bagasse silica nanoparticles
MWCNTs: Multi-walled carbon nanotubes
ROS: Reactive Oxygen species
DF: Drilling fluids
AFM: Atomic force microscope
CNF: Cellulose nanofibers
CNFSB: Sugarcane Bagasse cellulose nanofibers
CNC: Cellulose nanocrystals
MB: Methylene Blue
BPA: Bisphenol A
PVA: Polyvinyl alcohol
hLFCs: human lung fibroblast cells
TEMPO: 2,2,6,6-tetramethylpiperidine-1-oxyl
PEG: poly (ethylene glycol)
g-C_3_N_4_: graphitic carbon nitride
HCl: Hydrochloric acid
H_2_SO_4_: Sulphuric acid
SiO_2_: Silicon dioxide
Ag: Silver
AgCl: Silver chloride
Cu_2_O: Cuprous oxide
CuO: Cupric oxide
CdFe: Cadmium ferrite
ZF: Zinc fluoride
Na: Sodium
Ca: Calcium
Mg: Magnesium
Fe: Iron
K: Potassium
Mn: Manganese
Al: Aluminium
NaOH: Sodium hydroxide
E. Coli: *Escherichia coli*
*P. aeruginosa*: Pseudomonas aeruginosa
*S. aureus*: Staphylococcus aureus
*b. subtilis*: Bacillus subtilis
*K. planticola*: Klebsiella planticola
CdFe_2_O_4_: Cadmium ferrite
ZnFe_2_O_4_: Zinc ferrite
AgNO_3_: Silver nitrate
AgCl: Silver Chloride
Ag_2_O: Silver oxide
AlN: Aluminium nitride
Ca(OH)_2_: Calicum Hydroxide
Na_2_CO_3_: Sodium Carbonate
